# Cytotoxic, DNA Cleavage and Pharmacokinetic Parameter Study of Substituted Novel Furan C-2 Quinoline Coupled 1, 2, 4-Triazole and Its Analogs

**DOI:** 10.2174/1874104501812010073

**Published:** 2018-06-20

**Authors:** Rajpurohit Anantacharya, Nayak D. Satyanarayan, Bhuvanesh Sukhlal Kalal, Vinitha Ramanath Pai

**Affiliations:** 1 Department of Pharmaceutical Chemistry, Kuvempu University, Post Graduate Centre, Kadur, 577548, Chikkamagalur Dist, Karnataka, India; 2 Department of Biochemistry, Yenepoya Medical College, Yenepoya University, Mangaluru, 575018, Karnataka, India.; 3 Yenepoya Research Centre, Yenepoya University, Mangaluru, 575018, Karnataka, India.

## The Open Medicinal Chemistry Journal, **2018, 12: 60-72**

## The correct author list is mentioned below:

Rajpurohit Anantacharya^1^, Nayak D. Satyanarayan^1,*^, Bhuvanesh Sukhlal Kalal^2,3^ and Vinitha Ramanath Pai^2^

## The original list provided was:

Rajpurohit Anantacharya^1^, Nayak D. Satyanarayan^1,*^, Sukhlal K. Bhuvanesh^2,3^, Vinitha R. Pai^2^

